# Cross-cutting effect of type 2 diabetes on the sensorimotor control of balance

**DOI:** 10.3389/fcdhc.2024.1441947

**Published:** 2025-01-08

**Authors:** Trevor Lopatin, Ben Borngesser, Joshua Haworth

**Affiliations:** Department of Human Movement Science, Oakland University, Rochester, MI, United States

**Keywords:** diabetes, exercise, retinopathy, neuropathy, vestibular dysfunction, vestibulopathy

## Abstract

Type 2 Diabetes is a highly prevalent chronic disorder that affects multiple systems through microvascular complications. Complications such as diabetic peripheral neuropathy, diabetic retinopathy, and diabetic vestibular dysfunction (vestibulopathy) all directly interfere with the sensory components of balance and postural stability. The resulting impairments cause increased falls risk and instability, making it difficult to perform daily task or exercise. This commentary will provide clarity on the causes and relationship between the sensory complications of T2D, balance, and excise, while also providing recommendations and precautions for exercising with one of these sensory complications.

## 1 Introduction

Type 2 Diabetes (T2D) is a systemic disease that can have a far-reaching effect on daily life. The microvascular complications associated with T2D directly interfere with the sensory components of balance, increasing the risk for falls and injury. Exercise is very important for the management of T2D and can even help improve the symptoms of these microvascular complications, but precautions should be taken for those with complications to ensure their safety. This work will review the current literature to compile what is known about 1. The pathomechanics of T2D and the sensory complications caused by it, 2. How the sensorimotor control of balance is affected, 3. Proper exercise prescription and treatment options available for those with these sensory complications of T2D.

### 1.1 Sensorimotor control of balance

In a physical fitness-related setting, the ability to maintain, gain, or regain balance is crucial to completing tasks safely and effectively. This can also be defined as postural control (1). Postural control incorporates 3 different types of sensory information, including proprioception, vision, and vestibular (2). These sensory systems work together to provide postural feedback in order for the body to constantly reorganize and maintain balance (3). Each of these sensory components act as a fail safe for one another, but with the loss of each system an individual’s stability suffers. While balance loss is primarily seen as a physical impairment, it has also been shown to significantly affect psychological factors as well, such as increased fear of falling, denying independence as well as confidence in one’s ability to perform certain tasks (4).

### 1.2 Proprioception

Proprioception is important in serving as an afferent-efferent neurological pathway to maintain body stability and orientation (5). Within the muscles, tendons, and skin are receptors such as muscle spindles and cutaneous receptors that when stretched or forced upon are stimulated, providing positional information of the affected area (6, 7). The nervous system is responsible for relaying this information to the brain, any damage to these components may cause these signals to be altered and lead to injury through insufficient positional adjustments. During exercise the systems responsible for proprioception experience an increased demand as well as fatigue requiring accurate signaling to perform safely. Without proprioceptive neuromuscular function, the body struggles to “feel” destructive postural habits, as well as positional awareness of the affected limb (6, 8). This may lead to unsteady balance or overexertion of the muscle/limb, which may have significant consequences during exercise. Research within proprioception still needs more development in regard to mending proprioceptive deficits to improve function and overall balance (5).

### 1.3 Vision

The visual component of balance relays information about the ever-changing surrounding environment checking for accuracy of the movement and possible readjustments that need to be made (9). Studies have shown that when visual information is available the visual sense dominates the control of balance specifically during movement planning (10, 11). When visual information is not available other systems compensate for this loss of information causing decreased stability (12). Impairments to the visual system have a detrimental effect on postural stability, impacting the sensorimotor and vestibular-ocular reflex (13). When the vestibular-ocular reflex is damaged maintaining eye positioning during movement becomes disturbed, resulting in disorientation (14). As vision deteriorates a person’s field of vision may become blurred, distorted, or absent affecting their ability to see their surroundings and adapt to them.

### 1.4 Vestibular

The vestibular system works differently compared to the two prior senses, as its response is unaffected by the knowledge of the source and acts automatically through reflex (15). The vestibular system responds to acceleration of the head in space and therefore automatically signals self-motion. The three main structures related to balance are the semicircular canals, utricle, and saccule. All three of these are filled with fluid and contain small hairs that bend when the fluid shifts due to linear or rotational accelerations. These structures then send the information, via the vestibular nerve, to the brain (13). When any of these components become damaged, vestibular dysfunction is likely to appear resulting in damage to the vestibular-ocular reflex as well and instability (16).

## 2 Balance and exercise

Exercise relies heavily on the ability to maintain postural control, with the central nervous system playing a crucial role of integrating the sensory information for proprioceptive, visual, and vestibular senses (17). Through the utilization of these systems during exercise postural stability can also be enhanced through strengthening, adaptations, and compensations of these senses (18). Exercises such as Tai Chi, resistance/power training, three dimensional training, general physical activity, or computer based balance training have all been shown to improve postural stability (19). These improvements have been shown as a promising way of recovering lost sensation, reduced risk of falling, and increased physical function (19).

## 3 Balance and diabetes

**Figure 1:**
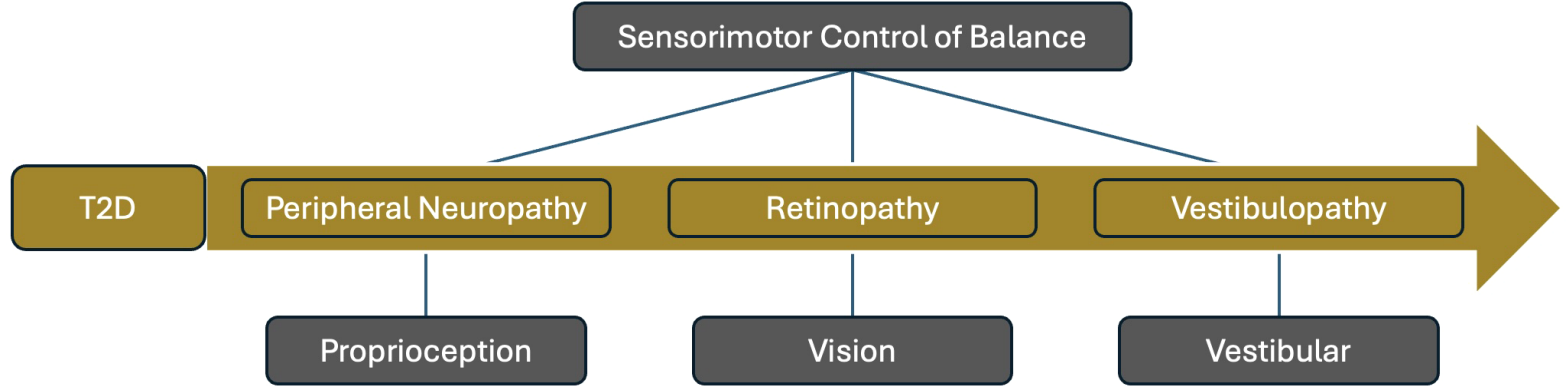
Diabetes sensory complications crosscut the sensorimotor control of balance by impacting proprioception, vision, and vestibular functions, the main sensory contributors to postural control and stability.

The sensorimotor component consists of the proprioception, vision, and vestibular senses (2, 20). When any of the 3 sensory components become impaired, the persons balance capabilities are reduced, leading to an increase in falls risk. While age is known as a large contributing factor to balance loss, studies have shown that person’s with T2D have increased balance impairment independent of age (21). The sensory complications of T2D (**Figure 1**), have the potential to affect all 3 of the sensorimotor components of balance. Neuropathy impacts proprioception (22), retinopathy impacts vision (23), and vestibular dysfunction impacts the vestibular system (24), making diabetics highly susceptible to balance impairments and falls (25, 26). This, alongside diabetics impaired ability to heal, makes minor injuries from falls much more dangerous for those with DM (27).

### 3.1 Diabetic peripheral neuropathy

Diabetic peripheral neuropathy (DPN) is one of the hall mark complications of T2D. Progression of DPN is often slow, with symptoms starting in the distal extremities first, where damage to the nerves of the feet and or hands cause increased sensitivity to pain, temperature, and eventually numbness (28). When left unmanaged or treated it can progress to more medial nerves, including those of the heart, resulting in cardiac autonomic neuropathy (CAN), which caused alterations to heart rate, blood pressure, and cardiac output (29). Screening for DPN is usually done using the monofilament test, sensory function tests, or symptomology of the peripheral extremities and Achilles tendon reflex (30). Further testing may be done to confirm diagnosis using a nerve conduction test to assesses electrical abnormalities in the nerve or a corneal confocal microscope to image the morphology of the nerve, though is not commonly used (31).

#### 3.1.1 Pathology of DPN

DPN is a slow progressive disease that is thought to be caused by the microvascular occlusion and hyperglycemia found in T2D (32). While the exact mechanism causing DPN is not yet well understood, there are several theories that provide some insight into the onset and progression of DPN. Aldose reductase (AR) is a regulatory enzyme found in the polyol pathway that is upregulated in ischemic hyperglycemic conditions (33). This pathway is responsible for converting glucose into fructose and produces advanced glycation end products (AGE) and sorbitol which are known to cause oxidative stress (34–36). This oxidative stress in turn causes structural changes in the peripheral nerve and to the dorsal root ganglion (33). It has been proposed that these structural changes are due to glycation, where AGE and sorbitol molecules are deposited into these nervous structures. AGE molecules induce oxidative stress and inflammation, which can directly harm nerve cells. Sorbitol, meanwhile, accumulates due to impaired glucose metabolism, leading to osmotic stress within Schwann cells. These factors contribute to toxicity, triggering apoptosis Schwann cells, demyelination, distal fiber degeneration, and impaired nerve regeneration (33, 35). The result of these changes can be seen in the nerves reduced conductive ability, resulting in altered or absence of signaling, where the affected limb may go completely numb.

#### 3.1.2 Relation to balance

The presence and severity of DPN have been shown to increase postural instability (37). Measures of balance in patients with DPN are limited to postural sway during quiet standing, resulting in increased postural sway (38). Postural movements during both quiet standing and walking have demonstrated greater variability in patients with DPN, which suggests an increased difficulty in regulating movement, destabilizing the body even more in itself (39). DPN most commonly leads to a decay in the lower extremities and nervous system, not allowing the important sensory systems to function properly (40). This creates a dearth of proprioceptive information coming from the lower extremities, resulting in postural instability during static on non-static situations (41). In the late stages with the development of CAN, patients may develop orthostatic hypotension, where sudden changes in body positioning lead to a drop in blood pressure and result in severe instability (29).

#### 3.1.3 Treatment for DPN

The treatments for DPN primarily focuses around reducing pain, where a wide variety of drugs may be prescribed. The anticonvulsant drug pregabalin is recommended by the American Academy of Neurology as first-line therapy, which inhibits neurotransmitters in the CNS helping to block pain (32). Antidepressants, analgesic, and opioids may also be used when necessary to help reduce pain (32). However, a downside of these pharmacological options is further reduction of proprioception, which exacerbates problems with sensorimotor control. Additional treatment options include BG, blood pressure, lipid and weight loss medications to help manage the symptoms and progression of the overarching disease, T2D.

#### 3.1.4 Exercise prescription

Recent studies suggested that aerobic physical activity, alone or in combination with resistance exercise, may be an effective therapeutic modality for Type 2 diabetes through revascularization and increased blood supply (42–44). It was found that a prescribed aerobic exercise regimen of mild intensity can positively influence both motor and sensory neuromuscular parameters (45). Diabetes patients are encouraged to do 30 minutes to an hour of aerobic activity most days of the week, as well as resistance training performed twice a week (46). More specific exercise programs such as sensory motor training or Tai Chi have been shown to improve neuromuscular structures and improve the symptoms of DPN (47). Specifically Tai chi has been found to increase vascular function and improve blood flow to the peripheral limbs (48). There is also a risk of those with DPN being unable to feel possible foot injuries and should take special care if one is present by only performing non weight bearing exercises such as swimming or biking (49).

### 3.2 Diabetic retinopathy

Diabetic Retinopathy (DR) is a prevalent complication of T2D that results in the loss of vision. DR is a progressive disease that stems from the microvascular damage and vascular occlusion in the retinas and is considered to have two clinical stages. The first stage, non-proliferative diabetic retinopathy (NPDR), occurs when vascular permeability, microaneurysms, and capillary occlusion are observable in the retinal vasculature (23, 50). The second stage, proliferative diabetic retinopathy (PDR), is more advanced stage that occurs with neovascularization, vitreous hemorrhage, or tractional retinal detachment (23, 50). During either of these stages diabetic macular edema (DME) may present and is “the most common cause of vision loss in patients with DR” (50). DR is diagnoses by a retinal eye exam, where an ophthalmologist will visually inspect the retina for lesions, vascular abnormalities, and edema in the retina (51).

#### 3.2.1 Pathology of DR

Hyperglycemia is responsible for the initial onset of NPDR, where elevated blood glucose levels cause retinal blood vessel dilation, pericyte apoptosis (cells providing structural support for capillaries), and endothelial cell apoptosis (52). With the loss of these pericyte cells, vascular permeability increases, microaneurysms formation, and breakdown of the blood-retinal barrier (BRB) in the capillaries of the retina. The onset of the second stage, PDR, is seen once progression pericyte and endothelial cell death leads to retinal ischemia (from capillary occlusion) and upregulation of vascular endothelial growth factor (VEGF) (53). VEGF is a strong angiogenic factor that causes increased vascular permeability and neovascularization (clinical feature of PDR) (54).

During these events, the increased permeability and breakdown of the BRB in the retinal capillaries causes fluid to accumulate in the macula (center of the retina), causing it to swell and thicken and eventually lead to DME and vision loss (50).

#### 3.2.2 Relation to balance

Retinopathy is well understood as contributing to gait instability and falls (13, 55). DR causes significant losses to a person’s visual sense, causing individuals (especially in the later stages) to not be able to accurately perceive and adjust to their environment (23). These losses can either be due to a person’s inability to receive the information (retinal damage) or accurately relay the information (ocular nerve damage) (56). With damage to the retina and the loss of visual information, greater reliance is put upon the other systems causing reduced postural stability. Loss of the peripheral visual field is another consequence of DR, that appears to cause a large effect on instability, specifically in side to side and forwards backwards movements (57). With damage to the ocular nerve the vestibular-ocular reflex becomes impaired, reducing eye tracking ability and ocular focusing, putting greater stress on the proprioception sense (14).

#### 3.2.3 Treatment of DR

There are several forms of treatment available for those with DR. The most promising are the anti-VEGF medications that have been found to reduce and even reverse DR (58, 59). This medication is injected monthly or bimonthly to help reduce VEGF levels in patients with DR. Intravitreal corticosteroids is a potent anti-inflammatory drug that is used to treat the early stages of DR (NPDR) and DME when patients do not respond to the anti-VEGF (50). Alongside medications, laser treatment is another option for treating DR. Laser photocoagulation has been found to reduce DME, prevent vision loss, and regress neovascularization, even though it is still unclear the exact mechanism behind this (50, 60).

#### 3.2.4 Exercise prescription

Exercise prescription for visually impaired patients can be difficult to provide due to situations where complications can outweigh expected benefit, especially in subjects with diabetes. Although exercise is beneficial in diabetes, previous studies have shown contradictory and inconclusive results on how it affects DR (61). Some studies have found that physical activity has been shown to slow the progression (62). Aerobic exercise has been shown to do this through reduced inflammation and oxidative stress in the eye (63). Careful monitoring of the intensity of the exercise is very important for those with DR. More vigorous forms of exercise, such as sprinting or heavy resistance training, may induce greater hemodynamic pressure and cause more ocular hemorrhaging, and thus should be avoided (64).

### 3.3 Vestibular apathy/dysfunction

Diabetic Vestibular dysfunction (DVD) is a prevalent complication of T2D that is not as well known (25). Studies have found that those with T2D are significantly more likely to have DVD compared to those without T2D (65). The vestibular system is a small complex of organs located in the inner ear that are responsible for relaying information about head orientation, accelerations, neural reflexes (66). When any of these organs or the vestibular nerve become damaged these senses and reflexes become impaired. There are several symptoms associated with DVD, such as vertigo, nausea, intolerance to head movement, and postural instability (67). Testing for DVD can be difficult due to the number of organs involved, with each requiring a different method to assess. The vestibulo-ocular reflex and the semicircular canals are assessed using a caloric test or video head impulse test, the vestibulo-spinal reflex and saccule can be assessed using a cVEMP, and the urticle is assessed using an ocular VEMP (24).

#### 3.3.1 Pathology of DVD

The exact pathology of T2D causing DVD is not yet well understood, but evidence from animal studies provides some insight into the possible mechanisms behind it. It is theorized that DVD can develop through hyperglycemias cascading effect on either the organs of the vestibular system or the vestibulocochlear nerve. The oxidative stress caused by chronic hyperglycemia was found to increase the extracellular matrix, lysosomes, and lipid droplets in the connective tissue of the saccule and utricle damaging the membrane and leading to impaired diffusion capabilities and ischemia (13, 68). This then leads to type 1 hair cell (cells involved in large head acceleration) degeneration in the saccule and utricle. With The vestibulocochlear nerve, elevated AGEs from chronic hyperglycemia induce demyelination and lysosomal digestion of the nerve, resulting in myelin sheath thinning and reduced axonal fiber diameters (13). Both of these pathways lead to dysfunction of the vestibular sense shown by “longer latency and reduced amplitude of vestibular evoked potentials” (13).

#### 3.3.2 Relation to balance

Postural control relies on many sensory cues from the vestibular system, specifically with accelerations and head positioning. This information is something that the other senses are not able to easily compensate for, causing heavy reliance on the proprioceptive and visual information available (69). DVD can either be unilateral or bilateral, without the presence of other sensory impairments, these two types of DVD have similar effects on a person’s postural stability, but once another sensory impairment is present, bilateral DVD becomes significantly more impactful (70). DVD has also been shown to have a multi-sense effect, with impairment of the vestibulo-ocular reflex also impacting a person’s ability to control eye movements and tracking (71, 72).

#### 3.3.3 Treatment of DVD

Currently there are no effective pharmacological or surgical treatment options available for those suffering with DVD. The only form of treatment that has shown some DVD related improvements is vestibular rehabilitation therapy (73). This exercise-based treatment program focuses on retraining aspects of a person’s motor control to compensate for the lack of vestibular input. Vestibular rehabilitation therapy has been shown to help alleviate symptoms such as dizziness and postural instability using specific balance exercises daily (73).

#### 3.3.4 Exercise prescription

As with the other sensory complications physical activity can act as a preventative measure to DVD but with its onset and its symptomatology, continuing exercise may become difficult and more dangerous (74). Vestibular rehabilitation exercises have been developed and can be utilized to train and compensate the vestibular sense with those experiencing DVD safely (75). Balance retaining and goal-directed eye-head exercises have been found to significantly improve postural stability in those with DVD (76, 77). Head movements exercises such as head turns, head-trunk turns, and head walking turns have all been shown to help recalibrate the vestibulo-ocular reflex (78, 79). Active body movements such as walking with sharp turns and sit-to-stand has been shown to help with vestibulospinal regulation (75, 78, 79).

## 4 Conclusion

This work summarized the pathomechanics of T2D and its sensory complications, their impact on the sensorimotor control of balance and exercise, and treatment options based on the current literature. DPN, DR, and DVD directly impair proprioception, vision and the vestibular sense leading to a significantly higher risk of falls and loss of stability. While exercise is a key component to the management of T2D, special considerations must be taken to ensure patient safety when exercising with these complications. Improper exercise programming may lead to worsening of the conditions or injury. As such routine screening practices are needed for these complications to ensure proper treatment and exercise programing is prescribed. Development of better screening methods to improve adherence and detection of T2D complications will ensure that individuals with T2D are able to receive the care and treatments they need.

## Data Availability

The original contributions presented in the study are included in the article/supplementary material. Further inquiries can be directed to the corresponding author.

## References

[1] Pollock AS Durward BR Rowe PJ Paul JP. What is balance? Clin. Rehabil. (2000) 14:402–6. doi: 10.1191/0269215500cr342oa 10945424

[2] Peterka RJ. Sensorimotor integration in human postural control. J. Neurophysiology. (2002) 88:1097–118. doi: 10.1152/jn.2002.88.3.1097 12205132

[3] Peterka RJ. Chapter 2 - Sensory integration for human balance control. In: Day BL Lord SR, editors. Handb Clin Neurol. (2018). 159:27–42. doi: 10.1016/B978-0-444-63916-5.00002-1 30482320

[4] Ray CT Horvat M Croce R Christopher Mason R Wolf SL. The impact of vision loss on postural stability and balance strategies in individuals with profound vision loss. Gait Posture. (2008) 28:58–61. doi: 10.1016/j.gaitpost.2007.09.010 18023185

[5] Laskowski ER Newcomer-Aney K Smith J. Proprioception. Phys. Med. Rehabil. Clinics North America. (2000) 11:323–40. 10810764

[6] Proske U. Exercise, fatigue and proprioception: a retrospective. Exp. Brain Res. (2019) 237:2447–59. doi: 10.1007/s00221-019-05634-8 31471677

[7] Collins DF. Proprioception: role of cutaneous receptors. In: Binder MD Hirokawa N Windhorst U, editors. Encyclopedia of neuroscience. Springer, Berlin, Heidelberg (2009). p. 3311–5. doi: 10.1007/978-3-540-29678-2_4825

[8] Cole J Waterman I. Pride and a daily marathon. 1st MIT Press ed. Cambridge, Mass: MIT Press (1995). 220 p.

[9] Goodman R Tremblay L. Using proprioception to control ongoing actions: dominance of vision or altered proprioceptive weighing? Exp. Brain Res. (2018) 236:1897–910. doi: 10.1007/s00221-018-5258-7 29696313

[10] Nieto-Guisado A Solana-Tramunt M Marco-Ahulló A Sevilla-Sánchez M Cabrejas C Campos-Rius J. The mediating role of vision in the relationship between proprioception and postural control in older adults, as compared to teenagers and younger and middle-aged adults. Healthcare. (2022) 10:103. doi: 10.3390/healthcare10010103 35052267 PMC8776119

[11] Bagesteiro LB Sarlegna FR Sainburg RL. Differential influence of vision and proprioception on control of movement distance. Exp. Brain Res. (2006) 171:358–70. doi: 10.1007/s00221-005-0272-y PMC10710692 16307242

[12] Schmidt D Carpes FP Milani TL Germano AMC. Different visual manipulations have similar effects on quasi-static and dynamic balance responses of young and older people. PeerJ. (2021) 9:e11221. doi: 10.7717/peerj.11221 34026347 PMC8121054

[13] D’Silva LJ Lin J Staecker H Whitney SL Kluding PM. Impact of diabetic complications on balance and falls: contribution of the vestibular system. Phys. Ther. (2016) 96:400–9. doi: 10.2522/ptj.20140604 PMC4774386 26251477

[14] Somisetty S Das JM. Neuroanatomy, vestibulo-ocular reflex. In: StatPearls. StatPearls Publishing, Treasure Island (FL (2024). Available at: http://www.ncbi.nlm.nih.gov/books/NBK545297/. 31424881

[15] Guerraz M Day BL. Expectation and the vestibular control of balance. J. Cogn. Neurosci. (2005) 17:463–9. doi: 10.1162/0898929053279540 15814005

[16] Hain TC Cherchi M Yacovino DA. Bilateral vestibular loss. Semin. Neurol. (2013) 33:195–203. doi: 10.1055/s-0033-1354597 24057822

[17] Shibata D. Improvement of dynamic postural stability by an exercise program. Gait Posture. (2020) 80:178–84. doi: 10.1016/j.gaitpost.2020.05.044 32521472

[18] Wang H Fan Z Liu X Zheng J Zhang S Zhang S. Effect of progressive postural control exercise versus core stability exercise in young adults with chronic low back pain: A randomized controlled trial. Pain Ther. (2023) 12:293–308. doi: 10.1007/s40122-022-00458-x 36454387 PMC9845492

[19] Howe TE Rochester L Neil F Skelton DA Ballinger C. Exercise for improving balance in older people. Cochrane Database Systematic Rev. (2011) 11. doi: 10.1002/14651858.CD004963.pub3/abstract PMC11493176 22071817

[20] Goble DJ Brar H Brown EC Marks CR Baweja HS. Normative data for the Balance Tracking System modified Clinical Test of Sensory Integration and Balance protocol. Med. Devices (Auckl). (2019) 12:183–91. doi: 10.2147/MDER.S206530 PMC6519013 31191047

[21] Deshpande N Hewston P Aldred A. Sensory functions, balance, and mobility in older adults with type 2 diabetes without overt diabetic peripheral neuropathy: A brief report. J. Appl. Gerontol. (2017) 36:1032–44. doi: 10.1177/0733464815602341 26324522

[22] Li L Zhang S Dobson J. The contribution of small and large sensory afferents to postural control in patients with peripheral neuropathy. J. Sport Health Science. (2019) 8:218–27. doi: 10.1016/j.jshs.2018.09.010 PMC6523875 31193300

[23] Shukla UV Tripathy K. Diabetic retinopathy. In: StatPearls. StatPearls Publishing, Treasure Island (FL (2024). Available at: http://www.ncbi.nlm.nih.gov/books/NBK560805/. 32809640

[24] Piker EG Romero DJ. Diabetes and the vestibular system. Semin. Hear. (2019) 40:300–7. doi: 10.1055/s-0039-1697032 PMC6785312 31602093

[25] Agrawal Y Carey JP Della Santina CC Schubert MC Minor LB. Diabetes, vestibular dysfunction, and falls: analyses from the National Health and Nutrition Examination Survey. Otol. Neurotol. (2010) 31:1445–50. doi: 10.1097/MAO.0b013e3181f2f035 20856157

[26] Lopatin T Ko M Brown E Goble D Haworth J. Normative percentile ranking best reveals sensorimotor impairments of postural sway in type 2 diabetes. Res. Methods Med. Health Sci. (2024), 1–7. doi: 10.1177/26320843241235582

[27] Dasari N Jiang A Skochdopole A Chung J Reece EM Vorstenbosch J. Updates in diabetic wound healing, inflammation, and scarring. Semin. Plast. Surg. (2021) 35:153–8. doi: 10.1055/s-0041-1731460 PMC8432997 34526862

[28] St. Onge EL Miller SA. Pain associated with diabetic peripheral neuropathy. P T. (2008) 33:166–76. PMC2730085 19750158

[29] Pop-Busui R. Cardiac autonomic neuropathy in diabetes. Diabetes Care. (2010) 33:434–41. doi: 10.2337/dc09-1294 PMC2809298 20103559

[30] Wang F Zhang J Yu J Liu S Zhang R Ma X. Diagnostic accuracy of monofilament tests for detecting diabetic peripheral neuropathy: A systematic review and meta-analysis. J. Diabetes Res. (2017) 2017:8787261. doi: 10.1155/2017/8787261 29119118 PMC5651135

[31] Yu Y. Gold standard for diagnosis of DPN. Front. Endocrinol. (Lausanne). (2021) 12:719356. doi: 10.3389/fendo.2021.719356 34764937 PMC8576350

[32] Cohen K Shinkazh N Frank J Israel I Fellner C. Pharmacological treatment of diabetic peripheral neuropathy. P T. (2015) 40:372–88. PMC4450668 26045647

[33] Yagihashi S Mizukami H Sugimoto K. Mechanism of diabetic neuropathy: Where are we now and where to go? J. Diabetes Investig. (2011) 2:18–32. doi: 10.1111/j.2040-1124.2010.00070.x PMC4008011 24843457

[34] Chung SSM Ho ECM Lam KSL Chung SK. Contribution of polyol pathway to diabetes-induced oxidative stress. J. Am. Soc. Nephrol. (2003) 14:S233. doi: 10.1097/01.ASN.0000077408.15865.06 12874437

[35] Akamine T Kusunose N Matsunaga N Koyanagi S Ohdo S. Accumulation of sorbitol in the sciatic nerve modulates circadian properties of diabetes-induced neuropathic pain hypersensitivity in a diabetic mouse model. Biochem. Biophys. Res. Commun. (2018) 503:181–7. doi: 10.1016/j.bbrc.2018.05.209 29864425

[36] Srivastava B Sen S Bhakta S Sen K. Effect of caffeine on the possible amelioration of diabetic neuropathy: A spectroscopic study. Spectrochimica Acta Part A: Mol. Biomolecular Spectroscopy. (2022) 264:120322. doi: 10.1016/j.saa.2021.120322 34509062

[37] Boucher P Teasdale N Courtemanche R Bard C Fleury M. Postural stability in diabetic polyneuropathy. Diabetes Care. (1995) 18:638–45. doi: 10.2337/diacare.18.5.638 8586001

[38] Bonnet CT Ray C. Peripheral neuropathy may not be the only fundamental reason explaining increased sway in diabetic individuals. Clin. Biomechanics. (2011) 26:699–706. doi: 10.1016/j.clinbiomech.2011.03.004 21458121

[39] Menz HB Lord SR St George R Fitzpatrick RC. Walking stability and sensorimotor function in older people with diabetic peripheral neuropathy1. Arch. Phys. Med. Rehabilitation. (2004) 85:245–52. doi: 10.1016/j.apmr.2003.06.015 14966709

[40] Cimbiz A Cakir O. Evaluation of balance and physical fitness in diabetic neuropathic patients. J. Diabetes its Complications. (2005) 19:160–4. doi: 10.1016/j.jdiacomp.2004.06.005 15866062

[41] Ghanavati T Shaterzadeh Yazdi MJ Goharpey S Arastoo AA. Functional balance in elderly with diabetic neuropathy. Diabetes Res. Clin. Practice. (2012) 96:24–8. doi: 10.1016/j.diabres.2011.10.041 22129655

[42] Castaneda C Layne JE Munoz-Orians L Gordon PL Walsmith J Foldvari M. A randomized controlled trial of resistance exercise training to improve glycemic control in older adults with type 2 diabetes. Diabetes Care. (2002) 25:2335–41. doi: 10.2337/diacare.25.12.2335 12453982

[43] Dunstan DW Daly RM Owen N Jolley D De Courten M Shaw J. High-intensity resistance training improves glycemic control in older patients with type 2 diabetes. Diabetes Care. (2002) 25:1729–36. doi: 10.2337/diacare.25.10.1729 12351469

[44] Maiorana A O’Driscoll G Goodman C Taylor R Green D. Combined aerobic and resistance exercise improves glycemic control and fitness in type 2 diabetes. Diabetes Res. Clin. Pract. (2002) 56:115–23. doi: 10.1016/s0168-8227(01)00368-0 11891019

[45] Balducci S Iacobellis G Parisi L Di Biase N Calandriello E Leonetti F. Exercise training can modify the natural history of diabetic peripheral neuropathy. J. Diabetes its Complications. (2006) 20:216–23. doi: 10.1016/j.jdiacomp.2005.07.005 16798472

[46] Borhade MB Singh S. Diabetes and exercise. In: StatPearls. StatPearls Publishing, Treasure Island (FL (2024). Available at: http://www.ncbi.nlm.nih.gov/books/NBK526095/. 30252351

[47] Streckmann F Zopf EM Lehmann HC May K Rizza J Zimmer P. Exercise intervention studies in patients with peripheral neuropathy: A systematic review. Sports Med. (2014) 44:1289–304. doi: 10.1007/s40279-014-0207-5 24927670

[48] Arce-Esquivel A Ballard J Haas B Hermanns M Rizer C Kimmel G. Effect of tai chi on vascular function among patients with peripheral neuropathy. Nursing Faculty Publications and Presentations (2016). Nursing Faculty Publications and Presentations. Paper 13. Available at: http://hdl.handle.net/10950/608.

[49] American Diabetes Association. Standards of medical care in diabetes—2014. Diabetes Care. (2013) 37:S14–80. doi: 10.2337/dc14-S014 24357209

[50] Wang W Lo ACY. Diabetic retinopathy: pathophysiology and treatments. Int. J. Mol. Sci. (2018) 19:1816. doi: 10.3390/ijms19061816 29925789 PMC6032159

[51] Sussman EJ Tsiaras WG Soper KA. Diagnosis of diabetic eye disease. JAMA. (1982) 247:3231–4. 7087063

[52] Romeo G Liu WH Asnaghi V Kern TS Lorenzi M. Activation of nuclear factor-kappaB induced by diabetes and high glucose regulates a proapoptotic program in retinal pericytes. Diabetes. (2002) 51:2241–8. doi: 10.2337/diabetes.51.7.2241 12086956

[53] Antonetti DA Barber AJ Hollinger LA Wolpert EB Gardner TW. Vascular endothelial growth factor induces rapid phosphorylation of tight junction proteins occludin and zonula occluden 1. A potential mechanism for vascular permeability in diabetic retinopathy and tumors. J. Biol. Chem. (1999) 274:23463–7. doi: 10.1074/jbc.274.33.23463 10438525

[54] Duffy AM Bouchier-Hayes DJ Harmey JH. Vascular endothelial growth factor (VEGF) and its role in non-endothelial cells: autocrine signaling by VEGF. In: Madame curie bioscience database. Austin (TX): Landes Bioscience (2000-2013). Available at: https://www.ncbi.nlm.nih.gov/books/NBK6482/.

[55] Ivers RQ Cumming RG Mitchell P Peduto AJ. Diabetes and risk of fracture: the blue mountains eye study. Diabetes Care. (2001) 24:1198–203. doi: 10.2337/diacare.24.7.1198 11423502

[56] Petersen KF Shulman GI. Pathogenesis of skeletal muscle insulin resistance in type 2 diabetes mellitus. Am. J. Cardiol. (2002) 90:11G–8G. doi: 10.1016/S0002-9149(02)02554-7 12231074

[57] Piras A Perazzolo M Scalinci SZ Raffi M. The effect of diabetic retinopathy on standing posture during optic flow stimulation. Gait Posture. (2022) 95:242–8. doi: 10.1016/j.gaitpost.2020.10.020 33781660

[58] Diabetic Retinopathy Clinical Research Network Wells JA Glassman AR Ayala AR Jampol LM Aiello LP. Aflibercept, bevacizumab, or ranibizumab for diabetic macular edema. N Engl. J. Med. (2015) 372:1193–203. doi: 10.1056/NEJMoa1414264 PMC4422053 25692915

[59] National Eye Institute. (2024). Diabetic retinopathy, National Eye Institute. Available at: https://www.nei.nih.gov/learn-about-eye-health/eye-conditions-and-diseases/diabetic-retinopathy (Accessed December 10, 2024).

[60] Hamdy O Goodyear LJ Horton ES. DIET AND EXERCISE IN TYPE 2 DIABETES MELLITUS. Endocrinol. Metab. Clinics. (2001) 30:883–907. doi: 10.1016/s0889-8529(05)70220-6 11727404

[61] Soleimani A Soltani P Karimi H Mirzaei M Esfahanian F Yavari M. The effect of moderate-intensity aerobic exercise on non-proliferative diabetic retinopathy in type II diabetes mellitus patients: A clinical trial. Microvascular Res. (2023) :149:104556. doi: 10.1016/j.mvr.2023.104556 37269942

[62] Tikkanen-Dolenc H Wadén J Forsblom C Harjutsalo V Thorn LM Saraheimo M. Frequent physical activity is associated with reduced risk of severe diabetic retinopathy in type 1 diabetes. Acta Diabetol. (2020) 57:527–34. doi: 10.1007/s00592-019-01454-y PMC7160093 31749048

[63] Ren C Liu W Li J Cao Y Xu J Lu P. Physical activity and risk of diabetic retinopathy: a systematic review and meta-analysis. Acta Diabetol. (2019) 56:823–37. doi: 10.1007/s00592-019-01319-4 30900027

[64] Graham C Lasko-M P. Exercise options for persons with diabetic complications. Diabetes Educ. (1990) 16:212–20. doi: 10.1177/014572179001600312 2185007

[65] Agrawal Y Carey JP Della Santina CC Schubert MC Minor LB. Disorders of balance and vestibular function in US adults: data from the National Health and Nutrition Examination Survey, 2001-2004. Arch. Intern. Med. (2009) 169:938–44. doi: 10.1001/archinternmed.2009.66 19468085

[66] Casale J Browne T Murray IV Gupta G. Physiology, vestibular system. In: StatPearls. StatPearls Publishing, Treasure Island (FL (2023). Available at: http://www.ncbi.nlm.nih.gov/books/NBK532978/. 30422573

[67] Dougherty JM Carney M Hohman MH Emmady PD. Vestibular dysfunction. In: StatPearls. StatPearls Publishing, Treasure Island (FL (2023). Available at: http://www.ncbi.nlm.nih.gov/books/NBK558926/. 32644352

[68] Myers SF Ross MD. Morphological evidence of vestibular pathology in long-term experimental diabetes mellitus. II. Connective tissue and neuroepithelial pathology. Acta Otolaryngol. (1987) 104:40–9. doi: 10.3109/00016488709109045 3499049

[69] Zacharias GL Young LR. Influence of combined visual and vestibular cues on human perception and control of horizontal rotation. Exp. Brain Res. (1981) 41:159–71. doi: 10.1007/BF00236605 6970678

[70] Herssens N Verbecque E McCrum C Meijer K van de Berg R Saeys W. A systematic review on balance performance in patients with bilateral vestibulopathy. Phys. Ther. (2020) 100:1582–94. doi: 10.1093/ptj/pzaa083 32367131

[71] Cullen KE. The vestibular system: multimodal integration and encoding of self-motion for motor control. Trends Neurosci. (2012) 35:185–96. doi: 10.1016/j.tins.2011.12.001 PMC4000483 22245372

[72] Goldberg JM Cullen KE. Vestibular control of the head: possible functions of the vestibulocollic reflex. Exp. Brain Res. (2011) 210:331–45. doi: 10.1007/s00221-011-2611-5 PMC4157641 21442224

[73] Han BI Song HS Kim JS. Vestibular rehabilitation therapy: review of indications, mechanisms, and key exercises. J. Clin. Neurol. (2011) 7:184–96. doi: 10.3988/jcn.2011.7.4.184 PMC3259492 22259614

[74] Morimoto H Asai Y Johnson EG Koide Y Niki J Sakai S. Objective measures of physical activity in patients with chronic unilateral vestibular hypofunction, and its relationship to handicap, anxiety and postural stability. Auris Nasus Larynx. (2019) 46:70–7. doi: 10.1016/j.anl.2018.06.010 30691599

[75] Han BI. Vestibular rehabilitation therapy: review of indications, mechanisms, and key exercises. In: Han BI, editor. Simplified vestibular rehabilitation therapy. Springer, Singapore (2021). p. 1–16. doi: 10.1007/978-981-15-9869-2_1

[76] Szturm T Ireland DJ Lessing-Turner M. Comparison of different exercise programs in the rehabilitation of patients with chronic peripheral vestibular dysfunction. J. Vestib Res. (1994) 4:461–79. doi: 10.3233/VES-1994-4606 7850042

[77] Herdman SJ Hall CD Schubert MC Das VE Tusa RJ. Recovery of dynamic visual acuity in bilateral vestibular hypofunction. Arch. Otolaryngology–Head Neck Surgery. (2007) 133:383–9. doi: 10.1001/archotol.133.4.383 17438254

[78] Keim RJ Cook M Martini D. How i do it: Otology and neurotology: A specific issue and its solution: Balance rehabilitation therapy. Laryngoscope. (1992) 102:1302–7. doi: 10.1288/00005537-199211000-00019 1405995

[79] Krebs DE Gill-Body KM Riley PO Parker SW. Double-blind, placebo-controlled trial of rehabilitation for bilateral vestibular hypofunction: Preliminary report. Otolaryngology–Head Neck Surgery. (1993) 109:735–41. doi: 10.1177/019459989310900417 8233513

